# Comprehensive analysis of bone tissue in extraction sockets of third molars after leukocyte and platelet rich fibrin and photobiomodulation applications

**DOI:** 10.1007/s00784-024-05872-3

**Published:** 2024-08-13

**Authors:** Sevgi Ozan Demirok, Cennet Neslihan Eroglu, Alaettin Koc

**Affiliations:** 1 Department of Oral and Maxillofacial Surgery, Faculty of Dentistry, Akdeniz University, Antalya, Turkey; 2 Department of Oral and Maxillofacial Radiology, Faculty of Dentistry, Yuzuncu Yil University, Van, Turkey; 3 Akdeniz Universitesi, Dis Hekimligi Fakultesi, Dumlupinar Bulvari, 07058 Turkey

**Keywords:** Photobiomodulation, L-PRF, Bone, Soft tissue

## Abstract

**Objectives:**

The aim of this study was to compare leukocyte and platelet-rich fibrin (L-PRF) and photobiomodulation (PBM) applications, which have been repeatedly reported to be superior to control groups, in terms of pain, soft tissue and bone healing in tooth extraction sockets.

**Materials and methods:**

This double-blind, randomized clinical study was completed with 34 patients, who had an indication for extraction of their bilaterally impacted teeth. The right and left teeth of the patients were randomly divided into L-PRF and PBM groups. L-PRF group was treated with the blood product centrifuged for 12 min at 2700 rpm, and the PBM group was treated with a diode laser at different points for 60 s with a wavelength of 940 nm in repeated sessions. Postoperative pain was evaluated using Visual Analogue Scale (VAS), soft tissue healing with Landry Index (LI), tissue healing in the distal region of mandibular second molar by probing depth measurement, and bone healing via panoramic x-ray using the Image J program.

**Results:**

No statistically significant difference was found for any variable compared between the groups.

**Conclusion:**

L-PRF and PBM applications provide similar support in the healing of extraction sockets. Nevertheless, the advantages and disadvantages of both methods determine their usage areas.

**Clinical relevance:**

While L-PRF is advantageous in the early healing of extraction sockets, PBM may be preferred in terms of bone trabeculation in the long term.

## Introduction

Alveolar wound healing after tooth extraction is a complex repair mechanism involving different tissue types such as bone and epithelium [1]. Healing mechanism of the extraction socket involves the formation and maturation of the blood clot, infiltration of fibroblasts to replace the coagulum, temporary matrix and reticular bone formation, and finally the formation of lamellar bone and bone marrow [2]. Within this healing physiology, searches continue to accelerate the process up to soft tissue epithelialization and replacement of mature bone tissue.

Leukocyte and platelet-rich fibrin (L-PRF) is an optimized clot with a strong fibrin structure and three-dimensional cell distribution (platelets, leukocytes and other circulating cells) [3]. L-PRF is more useful than other platelet concentrates due to its dense fibrin matrix structure [3, 4]. Fibrinogen present in L-PRF originates from 12 fibrin found in the blood and α granules released from platelets. Therefore, rate of fibrinogen is much higher than the other platelet concentrates [5]. Dohan et al. reported that growth factors such as transforming growth factor-beta (TGF-β), vascular endothelial growth factor (VEGF) and platelet-derived growth factor-AB (PDGF-AB) were released slowly from L-PRF for 7 days. During this period, the presence of high concentrations of growth factors in the environment allows L-PRF to stimulate the environment where wound healing occurs. The products contained in this natural fibrin material have high impact potential during the wound healing period [6]. In some recent studies, there are authors who argue that PRF concentrates do not make a significant difference in the healing of extraction sockets [7–9]. Generally speaking, the majority of studies in the literature indicate that PRF concentrates provide positive support for alveolar bone healing [10–15]. Photobiomodulation (PBM) is based on the absorption of visible red and near infrared light by photoreceptors in the cell (especially receptors in the electron transport chain within the mitochondrial membrane) [16]. In studies targeting bone healing with photobiomodulation, it has been proven that angiogenesis and connective tissue formation are more pronounced and bone formation is more advanced [17, 18]. Recently, numerous studies have been continuing on the stimulating effects of PBM on bone formation, and they have shown that PBM has a promoting effect on bone mineralization and the number of osteoblast cells [18, 19]. Photobiomodulation applications have become popular in the literature as a new trend for the preservation of bone tissue after extraction. There are also studies, albeit very limited, that conclude that PBM has no effect on bone healing after tooth extraction [20, 21]. However, in these studies, issues such as the time of application of the sessions, the number of sessions, whether the wavelength used for bone tissue penetration is sufficient, and the appropriateness of the times when bone density is examined carry question marks.

The aim of this split-mouth study was to compare the effects of L-PRF and PBM on pain, soft tissue and bone tissue healing after mandibular third molar extraction.

## Materials and methods

The research was carried out on healthy individuals aged between 18 and 40 years, recruited from the Oral and Maxillofacial Surgery Outpatient clinic. Eligible participants presented with bilateral mesioangular, similarly positioned, impacted wisdom teeth with retained bone, and were indicated for the extraction of these teeth.

Procedures were explained to the participants, and all of them signed consent forms before participating in the study. The study was approved by the Clinical Researches Ethics Committee of Faculty of Medicine of the University (08.04.2020/302). All procedures were performed in conformity with the 1964 Helsinki Declaration and its later amendments. The study was registered clinical trial registry retrospectively, number NCT06328413. And follows the recommendations of the CONSORT 2010 statement for reporting randomized trials.

In the power analysis, a sample size of 32 participants was determined to be necessary to achieve a power of 90% with a margin of error of 0.05.

The study included American Society of Anesthesiologists (ASA) Class-I volunteers between 18 and 40 years of age who had wisdom teeth (Pell and Gregory Class II, Position B) with bone retention in bilateral symmetrical position with an indication for extraction, and had second molars in the mouth, and did not use steroids or anti-inflammatory drugs in the last 3 months. Patients with pericoronitis, smoking habits, active periodontal disease, those who were pregnant, breastfeeding, or were unable to give personal consent, and those with missing physical examination and follow-up records were excluded.

**Fig. 1:**
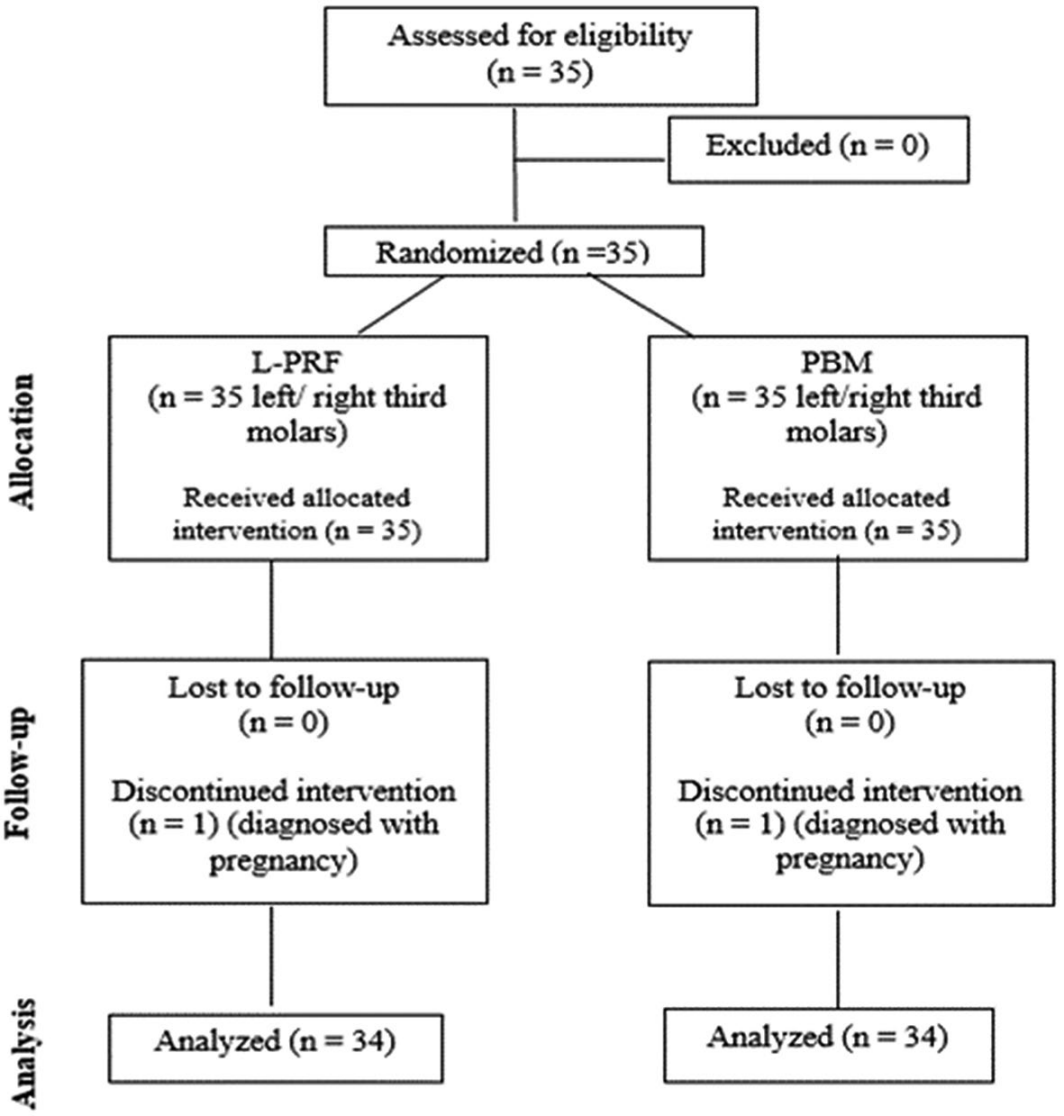
Flow diagram of study of the single centered parallel randomized group

The study groups included the right and left side teeth of the same patient, and simple randomization method was used to determine the side that would be included in the L-PRF and PBM groups (Figure-1).

### Surgical procedure

The operations were performed by a single oral and maxillofacial surgeon (S.O.D) under local anesthesia using 80 mg Articaine Hydrochloride with 0.02 mg epinephrine. Inferior alveolar and buccal nerve anesthesia was achieved with local anesthesia. Thereafter, a horizontal incision was made with a No. 15 scalpel and a vertical relaxing incision was made from the mesiobuccal of the lower second molar tooth, and the mucoperiosteal flap was removed. After that, the bone covering the tooth was removed with a round bur under physiological saline irrigation and the tooth was divided and removed. After bleeding control was achieved, 10 ml of blood that was taken from the patient shortly before the extraction was centrifuged in a glass-covered anticoagulant-free plastic tube at 2700 rpm for 12 min and the L-PRF obtained was placed in the extraction socket. L-PRF was not placed on the other side. Both operation areas were closed with 3.0 silk sutures, provided that the flap was in its original position, and the stitches were removed 1 week after the operation.

After the operation, the patients were prescribed 1000 mg Amoxicillin twice a day for 5 days, Benzydamine HCL + Chlorhexidine Gluconate mouthwash 3 times a day for 5 days and 400 mg Etodolac 2 times a day for 5 days. Operation time was considered as the time from the moment the first incision was made to the moment the last suture was placed.

### Laser application

Photobiomodulation was applied intraorally from the occlusal and buccal regions to the tooth extraction socket on the side where L-PRF was not applied, on the 2nd, 4th, 7th, 11th, 14th, 18th and 21st postoperative days, at the values shown in Table-1.

**Table 1:** Details of photobiomodulation application

Manufacturer	Biolase Technology, Inc., Irvine, CA
**Model**	Biolase Epic X
**Year of manufacturing**	30.10.2018
**Type**	InGaAsP (Diode) laser
**Wavelength**	940 nm
**Pulse Mode**	Continuous
**Spot Size**	1 cm^2^
**Power Density**	0.5 W/cm^2^
**Irradiation Time**	60 s.
**Energy Density**	10 J/cm^2^
**Total energy (for each application)**	30 J
**Number of Irradiations**	2 points
**Irradiated area**	2 cm^2^
**Application site-Contact/noncontact**	Intraoral buccal (noncontact) + occlusal (noncontact))
**Number and frequency of applications**	7 times (on days 2,4,7,11,14,18 and 21)
**Total amount of energy**	60 J in one session (occlusal 30 J + buccal 30 J) Total 420 J

### Assessment of pain

Pain on the 2nd, 4th and 7th days after tooth extraction was evaluated with the Visual Analogue Scale (VAS)-pain. Patients were asked to evaluate the intensity of their pain on a scale of 0 to 10 for both sides separately (0 = no pain, 10 = unbearable pain). These values were recorded when the patients came to the laser application.

### Soft tissue evaluation

Soft tissue healing was evaluated by the Landry Healing Index [22] and by the probing depth in the adjacent tooth postoperatively at the end of the 1st and 2nd week, and 1st month.

The investigator collecting clinical data was blinded.

### Evaluation of bone tissue healing

For the comparison of bone tissue healing, an Orthopantomograph (OPG) was taken with a Planmeca Promax (Helsinki, Finland) device (66 kV 10 mA) in the 1st and 3rd months, and the bone density and trabecular pattern of the extraction sockets in the images opened in the Romexis program were examined and compared by the same radiologist blinded for the study groups using the Image J program.

To create a sample for bone density and trabecular pattern, bone density and trabeculation in the apical 1/3 and middle 1/3 parts of the extraction sockets were examined and their averages were taken.

In the program, 20 × 20 pixel squares were used as ‘Region of interest (ROI) areas, and the ROI areas in the tooth socket where laser was applied after the 3rd molar tooth extraction and in the root apical third and root middle third in the trabecular bone in the tooth socket on the opposite side where L-PRF was applied, were drawn manually using the ‘Rectangle’ section in the relevant software, on the panoramic radiographs taken in the 1st and 3rd months after tooth extraction. While measuring density, the bone values determined in the ROI were obtained by using the ‘Analyze’ section in the Image J software.

While calculating the Fractal Dimension (FD) in trabecular pattern measurement, the procedures were carried out in Image J software by following the steps of White and Rudolph [23]. Each selected ROI was duplicated after cutting. It was blurred with a Gaussian filter (sigma, 35) to eliminate large-scale variations in the duplicated image. In order to distinguish between bone marrow spaces and trabecular bone structures, 128 gray values were added after the blurred image was subtracted from the original ROI image. Afterwards, a two-color image (black and white) was obtained with the ‘binarization’ process. While the noise in the resulting image was eliminated by ‘erosion’, the outer contours of the structures were clarified by the ‘dilation’ process. With the ‘invert’ process, the black areas in the image reflected the trabecular bone while the white areas reflected the bone marrow cavities. The image was made ready for FD analysis with the ‘skeletonization’ process. The FD process was completed by calculating 2–64 pixel wide boxes in the skeletal image. ‘The ‘box-counting technique’ was applied with the ‘Fractal box count’ process in Image J software, and bone density was calculated with the “histogram” plugin in Image J software.

### Statistical analysis

The data obtained in this study were analyzed with the IBM SPSS Statistics Version 23 (Statistical Package for the Social Sciences, Chicago, IL) package program.

The assumption of normal distribution of variables was checked with the Shapiro-Wilk test. The difference between the groups was evaluated with the Mann Whitney-U test. In all analyses, the significance level was taken as 0.05.

## Results

A total of 34 patients, 26 women and 8 men, whose impacted lower third molars were surgically extracted, were included in the study. The average age of the patients participating in the study was 20.24 ± 2.54 years. The mean operation time was 10.41 ± 3.29 min on the L-PRF side, and 10.15 ± 3.16 min on the PBM side.

Although not statistically significant, it was observed that the mean pain level was lower in the L-PRF group on postoperative day 2 and with laser application after day 2, the mean pain level in the laser group was lower than in the L-PRF group (Table-2).

**Table 2:** Comparison of mean VAS-pain scores between the groups

VAS-pain scores	Groups	*n*	Mean ± SD	*p*
Day-2	L-PRF	34	3.65 ± 2.46	0.814
	PBM	34	3.74 ± 2.40	
Day-4	L-PRF	34	2.82 ± 2.17	0.606
	PBM	34	2.56 ± 2.09	
Day-7	L-PRF	34	1.12 ± 1.45	0.750
	PBM	34	1 ± 1.37	

Mann-Whitney U Test

Considering soft tissue healing, no statistically significant difference was found between the groups in terms of Landry Index score, and the probing depth of the distal tooth number 7 at the postoperative 1st and 2nd week, and 1st month (Table-3).

**Table 3:** Comparison of Landry Index scores and probing depth between the groups

Measurement	Time Point	Treatment Group	(*n*)	Mean ± SD	*p*-value
**Landry Index scores**	1st week	L-PRF	34	1.88 ± 0.59	0.788
		PBM	34	1.85 ± 0.61	
	2nd week	L-PRF	34	2.50 ± 0.62	0.596
		PBM	34	2.62 ± 0.74	
	1st month	L-PRF	34	3.41 ± 0.66	0.459
		PBM	34	3.50 ± 0.71	
**Probing Depth**	1st week	L-PRF	34	4.65 ± 2.19	0.841
		PBM	34	4.79 ± 2.36	
	2nd week	L-PRF	34	3.53 ± 2.16	0.895
		PBM	34	3.49 ± 2.22	
	1st month	L-PRF	34	2.53 ± 1.88	0.874
		PBM	34	2.29 ± 1.38	

Mann-Whitney U Test

In the comparison of bone tissue healing, though there was no statistically significant difference in density and trabeculation measurements between the two groups, the mean density values were found to be greater in the L-PRF group and the 3rd month mean FD value was found to be greater on the PBM side (Table-4).

**Table 4:** Comparison of mean fractal dimension and density values by groups

Radiographic Data	Groups	Mean ± SD	*p*
**Density (1st month)**	L-PRF	149.72 ± 28.14	0.469
	PBM	146.34 ± 28.50	
**FD (1st month)**	L-PRF	1090.41 ± 122.07	0.704
	PBM	1076.26 ± 96.59	
**Density (3rd month)**	L-PRF	158.37 ± 28.49	0.912
	PBM	156.96 ± 32.93	
**FD (3rd month)**	L-PRF	1019.76 ± 210.07	0.854
	PBM	1051.76 ± 150.06	

FD: Fractal dimension

Mann-Whitney U Test

## Discussion

PBM and L-PRF are two currently used methods in tissue engineering. We thought that these two methods should be compared in a split-mouth study and the results should be included in the literature. For this reason, the aim of this present study was to determine whether PBM and L-PRF applications, which have been shown to be effective against control groups many times before, can provide an advantage over each other in pain, bone healing, and soft tissue healing and pockets in the distal region of the adjacent tooth in extraction sockets. The results showed no significant difference in mean pain intensity on the 2nd, 4th and 7th postoperative days. However, it was observed clinically that the mean pain level was lower in the L-PRF group on the 2nd postoperative day, and in the PBM group on the 4th and 7th postoperative days. In the study by Ritto et al., the mean VAS-pain scores on the 1st, 3rd and 7th days after bilateral impacted tooth extraction were higher in the control group than that in the L-PRF group [15]. Similarly, in the bilateral impacted tooth extractions performed by Kapse et al., the mean VAS-pain scores were found to be significantly lower in the L-PRF group compared to the control group on days 1, 3, 7 and 14 [24]. In the study conducted by Hadad et al., the mean VAS-pain scores at the side on which PBM was applied were found to be significantly lower than the control side on which PBM was not applied after bilateral impacted tooth extraction (at 0, 12, 24, 48 and 72 h after PBM) [25]. In our study, while there was a clinically meaningful benefit in favor of L-PRF in the first 2 days, PBM became more advantageous as of the 4th day. Since pain intensity is generally higher in the first 2 days after tooth extraction, L-PRF may make a small difference in terms of pain in the early period.

There are many complications associated with the surgical removal of impacted third molars. One of the most overlooked is the formation of a deep periodontal pocket distal to the mandibular second molar after removal of an impacted mandibular third molar [26]. Kan et al., in their study measured pocket depths 6–36 months after surgery in 158 patients who had mandibular wisdom teeth extracted and found pocket depths of more than 5 mm in 67% of the adjacent second molars [27]. In Ritto et al.’s study, there was no significant difference between the distal pocket probing depth of the mandibular second molar in the L-PRF and control groups at the end of the 3rd month after extraction [15]. In our study, no significant difference was found in the 1st month probing depths between the two sides. The probing depth was not re-evaluated at the end of the 3rd month. There is a possibility that there may have been a difference in the 3rd month values between the groups. However, in that case, issues such as oral hygiene, plaque index, and the need to separately evaluate the periodontal status before extraction should be included [28]. While evaluating the effects of applications on pocket depth, we think that the evaluations in the 3rd month may be more misleading due to confounding factors.

Considering soft tissue healing, no statistically significant difference was found between the groups in the postoperative 1st week, 2nd week and 1st month. Consistent with the literature, PBM and L-PRF have been reported to have similar effects on soft tissue healing [29].

There is no definitive unity in determining the area to be examined on x-ray and different methods were used in the studies. Ritto et al. compared the smallest point where the axial, sagittal and coronal images of the socket intersect in cone beam computed tomography (CBCT) images [15]. In the average of the values in the apical, middle and cervical 1/3 areas of the socket in periapical x-rays were taken in the study of Varghese et al. [30]. In the study by Baslarli et al., extraction sockets were circled in the panoramic x-ray and density values were measured in the Image J program [31]. In our study, while determining the area to be examined in the x-ray, the cervical 1/3 area was not examined due to direct intra-oral contact and bone filling starting from the apical 1/3, and the average of the data of the apical and middle 1/3 was included in the analysis.

Thanks to fractal analysis (FA), the effects of various disorders on bone structures can be numerically predicted. Image J software is used in this method and there are current studies using ImageJ software to analyze bone quality [32]. Also, there are some reports validating this software in visualizing and interpreting biomedical images [33, 34]. The microarchitecture of trabecular bone can be assessed independently of bone density using a variety of two-dimensional or cross-sectional images. Microarchitectural changes in the jaws, which may occur earlier than linear changes, can be evaluated with the aforementioned analysis. When interpreting FA values, bone with a higher value is reported to have a more complex structure and less porosity [32].

Computed tomography is certainly the gold standard for bone evaluation. However, CT is both costly and causes the patient to receive a higher dose of radiation than panoramic x-ray. For these reasons, it has been observed that periapical x-rays and panoramic x-rays, which are more ethically appropriate, have been used in the studies [7, 30, 35].

In the literature, there are studies in which PRF and control groups were evaluated with the split mouth technique after impacted tooth extraction, and bone healing was found to be significantly better in the sockets on the side where PRF was applied [15, 24, 30, 31, 36]. In these studies, PRF groups were observed to be superior over the control groups, mostly in the 3rd month. In the present study, although there was no statistically significant difference in the 1st and 3rd month density and trabecular quality measurements between the two groups, the density values were found to be greater in the L-PRF group both at the 1st and 3rd months, and the FD value indicating the trabeculation quality has given better results on the PBM side at the 3rd month. According to these results, regardless of bone quality, L-PRF method stands out as more advantageous in the early and late periods of massive bone deposition. However, in terms of trabecular quality; while the L-PRF method is still advantageous in the early period, it is noteworthy that the trabecular quality is better in the PBM method in the late period of bone healing. Further prospective studies can be performed to analyze the great improvement in the bone quality with PBM over time.

Two different studies performed by Pereira et al. reported no difference between the two methods in terms of bone healing according to CT results when advanced platelet-rich fibrin (A-PRF) and PBM were compared with the control groups [8, 37]. In the study where A-PRF was applied, it was reported that while more bone density was found on the 7th day after maxillary 3rd molar extraction, no difference was observed at 90 days [8]. In the study where PBM was applied, PBM showed slightly better bone density than the control group on the 7th day after mandibular 3rd molar extraction, but no difference was observed at the end of 90 days [37]. We think that more than 2 sessions of PBM is needed to support bone healing. Also, the threshold energy value of bone tissue for PBM is not clear. The applied energy density may have been insufficient for bone tissue stimulation. While similar studies above only evaluated bone density, the fact that our study included fractal analysis reflecting trabecular quality highlights the “novelty” aspect of this study.

The absence of a control group in our study constitutes a limitation. However, considering the repeatedly demonstrated superiority of these two methods over a control group, establishing one may not have been meaningful for this study. Furthermore, the creation of a third group would introduce the limitation of mitigating individual response variations provided by the split-mouth design. Another potential limitation could have been the absence of a longer follow-up period. Nevertheless, as mentioned above, no significant differences between the experimental and control groups have been observed in any study after the third month. Therefore, the initial three-month recovery period has been considered for evaluation.

The PBM application could have been initiated on the day of the extraction. Evidence exists that PBM induces heat increase in the applied tissue, potentially reaching significant levels [38, 39]. Therefore, considering the possibility of increased bleeding and swelling on the day of the operation, the initial application was deferred. If it had been performed on the operation day, different results might have been observed for pain data on the second day and soft tissue healing at one week. However, we do not believe it would have made a significant difference for bone healing and subsequent data. There were several reasons for performing bilateral tooth extraction in the same session. The most important among these was to obtain results with the same period of healing potential as a metabolic response. In intermittent extraction, disadvantages such as the effect of menstrual cycle in female patients, potential change in pain threshold in the second procedure, increased x-ray exposure, and extended follow-up period leading to patient and consequently data loss directed us towards simultaneous extraction.

## Conclusion

Within the limitations of the study, we could not find any clinical study involving bone and soft tissue healing after extraction using the split mouth method for PBM and L-PRF. There was no statistically significant difference in the pain level, and soft and hard tissue healing between the two study groups. L-PRF and PBM applications provide similar support in the healing of extraction sockets. However, while L-PRF is advantageous in the early healing of extraction sockets, PBM may be preferred in terms of bone trabeculation in the long term. On the other hand, the disadvantage of L-PRF is that it is an invasive procedure, completing the procedure in a single session is a positive situation for patients with limited time. PBM is a non-invasive procedure, but repeated sessions are needed.

## Data Availability

No datasets were generated or analysed during the current study.

## References

[1] Mozzati M, Martinasso G, Cocero N, Pol R, Maggiora M, Muzio G, Canuto RA (2011) Influence of superpulsed laser therapy on healing processes following tooth extraction. Photomed Laser Surg 29(8):565–571. 10.1089/pho.2010.2921 21631375 10.1089/pho.2010.2921

[2] Park JB, Ahn SJ, Kang YG, Kim EC, Heo JS, Kang KL (2015) Effects of increased low-level diode laser irradiation time on extraction socket healing in rats. Lasers Med Sci 30(2):719–726. 10.1007/s10103-013-1402-6 23929563 10.1007/s10103-013-1402-6

[3] Dohan Ehrenfest DM, Del Corso M, Diss A, Mouhyi J, Charrier JB (2010) Three dimensional architecture and cell composition of a Choukroun’s platelet-rich fibrin clot and membrane. J Periodontol 81(4):546–555. 10.1902/jop.2009.090531 20373539 10.1902/jop.2009.090531

[4] Dohan DM, Choukroun J, Diss A, Dohan SL, Dohan AJ, Mouhyi J, Gogly B (2006) Platelet rich fibrin (PRF): a second-generation platelet concentrate. Part I: technological concepts and evolution. Oral Surg Oral Med Oral Pathol Oral Radiol Endod 101(3):e37–44. 10.1016/j.tripleo.2005.07.008 16504849 10.1016/j.tripleo.2005.07.008

[5] Dohan Ehrenfest DM, Rasmusson L, Albrektsson T (2009) Classification of platelet concentrates: from pure platelet-rich plasma (P-PRP) to leucocyte- and platelet-rich fibrin (L-PRF). Trends Biotechnol 27(3):158–167. 10.1016/j.tibtech.2008.11.009 19187989 10.1016/j.tibtech.2008.11.009

[6] Dohan DM, de Peppo GM, Doglioli P, Sammartino G (2009) Slow release of growth factors and thrombospondin-1 in Choukroun’s platelet-rich fibrin (PRF): a gold standard to achieve for all surgical platelet concentrates technologies. Growth Factors 27(1):63–69. 10.1080/08977190802636713 19089687 10.1080/08977190802636713

[7] Aravena PC, Sandoval SP, Pizarro FE, Simpson MI, Castro-Adams N, Serandour G, Rosas C (2021) Leukocyte and platelet rich fibrin have same effect as blood clot in the 3-diemensional alveolar ridge preservation. A split-mouth randomized clinical trial. J Oral Maxillofac Surg 79(3):575–584. 10.1016/j.joms.2020.10.006 33171113 10.1016/j.joms.2020.10.006

[8] Pereira DA, Mendes PGJ, Prisinoto NR, de Rezende Barbosa GL, Soares PBF, de Oliveira GJPL (2023) Advanced platelet-rich-fibrin (A-PRF +) has no additional effect on the healing of post-extraction sockets of upper third molars. A split mouth randomized clinical trial. Oral Maxillofac Surg 27(3):411–419. 10.1007/s10006-022-01075-w 35614276 10.1007/s10006-022-01075-w

[9] Abad CE, Sanz-Sanchez I, Serrano V, Sanz Esporrin J, Sanz-Martin I, Sanz M (2023) Efficacy of the application of leukocyte and platelet-rich fibrin (L-PRF) on alveolar ridge preservation. A randomized controlled clinical trial. Clin Implant Dent Relat Res 25(3):592–604. 10.1111/cid.13208 37088697 10.1111/cid.13208

[10] Whitman DH, Berry RL, Green DM (1997) Platelet gel: an autologous alternative to fibrin glue with applications in oral and maxillofacial surgery. J Oral Maxillofac Surg 55(11):1294–1299. 10.1016/s0278-2391(97)90187-7 9371122 10.1016/s0278-2391(97)90187-7

[11] Rodrigues ED, Pontual AD, Macedo RA, Nascimento E, Vasconcelos BC (2023) Evaluation of bone repair with platelet-rich fibrin following the extraction of impacted third molars-randomized clinical trial. Med Oral Patol Oral Cir Bucal 28(5):e433–e441. 10.4317/medoral.25856 37330965 10.4317/medoral.25856 PMC10499344

[12] Clark D, Rajendran Y, Paydar S, Ho S, Cox D, Ryder M, Dollard J, Kao RT (2018) Advanced platelet-rich fibrin and freeze-dried bone allograft for ridge preservation: a randomized controlled clinical trial. J Periodontol 89(4):379–387. 10.1002/JPER.17-0466 29683498 10.1002/JPER.17-0466 PMC6483085

[13] Canellas JVDS, da Costa RC, Breves RC, de Oliveira GP, Figueredo CMDS, Fischer RG, Thole AA, Medeiros PJD, Ritto FG (2020) Tomographic and histomorphometric evaluation of socket healing after tooth extraction using leukocyte- and platelet-rich fibrin: a randomized, single-blind, controlled clinical trial. J Craniomaxillofac Surg 48(1):24–32. 10.1016/j.jcms.2019.11.006 31810848 10.1016/j.jcms.2019.11.006

[14] Hauser F, Gaydarov N, Badoud I, Vazquez L, Bernard JP, Ammann P (2013) Clinical and histological evaluation of postextraction platelet-rich fibrin socket filling: a prospective randomized controlled study. Implant Dent 22(3):295–303. 10.1097/ID.0b013e3182906eb3 23644909 10.1097/ID.0b013e3182906eb3

[15] Ritto FG, Pimentel T, Canellas JVS, Junger B, Cruz M, Medeiros PJ (2019) Randomized double-blind clinical trial evaluation of bone healing after third molar surgery with the use of leukocyte- and platelet-rich fibrin. Int J Oral Maxillofac Surg 48(8):1088–1093. 10.1016/j.ijom.2019.01.020 30910410 10.1016/j.ijom.2019.01.020

[16] Walsh LJ (1997) The current status of low level laser therapy in dentistry. Part 1. Soft tissue applications. Aust Dent J 42(4):247–. 10.1111/j.1834-7819.1997.tb00129.x. 54 9316312 10.1111/j.1834-7819.1997.tb00129.x

[17] Khadra M, Kasem N, Haanaes HR, Ellingsen JE, Lyngstadaas SP (2004) Enhancement of bone formation in rat calvarial bone defects using low-level laser therapy. Oral Surg Oral Med Oral Pathol Oral Radiol Endod 97(6):693–700. 10.1016/j.tripleo.2003.11.008 15184850 10.1016/j.tripleo.2003.11.008

[18] Ribeiro LNS, de Figueiredo FAT, da Silva Mira PC, Arnez MFM, Matsumoto MAN, de Menezes LM, Küchler EC, Stuani MBS (2022) Low-level laser therapy (LLLT) improves alveolar bone healing in rats. Lasers Med Sci 37(2):961–969. 10.1007/s10103-021-03340-y 34002343 10.1007/s10103-021-03340-y

[19] Garavello-Freitas I, Baranauskas V, Joazeiro PP, Padovani CR, Dal Pai-Silva M, da Cruz-Höfling MA (2003) May-Jun;70(2):81 – 9 Low-power laser irradiation improves histomorphometrical parameters and bone matrix organization during tibia wound healing in rats. J Photochem Photobiol B 10.1016/s1011-1344(03)00058-7 10.1016/s1011-1344(03)00058-7 12849698

[20] Kucerova H, Dostalova T, Himmlova L, Bartova J, Mazanek J (2000) Low-level laser therapy after molar extraction. J Clin Laser Med Surg 18(6):309–315 11572225

[21] Paschoal MA, Santos-Pinto L (2012) Therapeutic effects of low-level laser therapy after premolar extraction in adolescents: a randomized double-blind clinical trial. Photomed Laser Surg 30(9):559–564. 10.1089/pho.2012.3243 22870960 10.1089/pho.2012.3243

[22] Landry RG, Turnbull RS, Howley T (1988) Effectiveness of benzydamine HCL in the treatment of periodontal post-surgical patients. Res Clin Forums 10:105–118

[23] White SC, Rudolph DJ (1999) Alterations of the trabecular pattern of the jaws in patients with osteoporosis. Oral Surg Oral Med Oral Pathol Oral Radiol Endod 88(5):628–635. 10.1016/s1079-2104(99)70097-1 10556761 10.1016/s1079-2104(99)70097-1

[24] Kapse S, Surana S, Satish M, Hussain SE, Vyas S, Thakur D (2019) Autologous platelet-rich fibrin: can it secure a better healing? Oral Surg Oral Med Oral Pathol Oral Radiol 127(1):8–18. 10.1016/j.oooo.2018.08.010 30287202 10.1016/j.oooo.2018.08.010

[25] Hadad H, Santos AFP, de Jesus LK, Poli PP, Mariano RC, Theodoro LH, Maiorana C, Souza FÁ (2022) Photobiomodulation Therapy improves Postoperative Pain and Edema in Third Molar surgeries: a Randomized, comparative, Double-Blind, and prospective clinical trial. J Oral Maxillofac Surg 80(1):37.e1-37.e12. 10.1016/j.joms.2021.08.267 10.1016/j.joms.2021.08.267 34656515

[26] Motamedi MH (1999) Preventing periodontal pocket formation after removal of an impacted mandibular third molar. J Am Dent Assoc 130(10):1482–1484. 10.14219/jada.archive.1999.0060 10570593 10.14219/jada.archive.1999.0060

[27] Kan KW, Liu JK, Lo EC, Corbet EF, Leung WK (2002) Residual periodontal defects distal to the mandibular second molar 6–36 months after impacted third molar extraction. J Clin Periodontol 29(11):1004–1011. 10.1034/j.1600-051x.2002.291105.x 12472993 10.1034/j.1600-051x.2002.291105.x

[28] Montero J, Mazzaglia G (2011) Effect of removing an impacted mandibular third molar on the periodontal status of the mandibular second molar. J Oral Maxillofac Surg 69(11):2691–2697. 10.1016/j.joms.2011.06.205 21864969 10.1016/j.joms.2011.06.205

[29] Mukhtar S, Bains VK, Chandra C, Srivastava R (2023) Evaluation of low-level laser therapy and platelet-rich fibrin on donor site healing after vascularized interpositional periosteal connective tissue flap: a randomized clinical study. Lasers Med Sci 38(1):68. 10.1007/s10103-023-03725-1 36752882 10.1007/s10103-023-03725-1 PMC9907210

[30] Varghese MP, Manuel S, Kumar LKS (2017) Potential for Osseous regeneration of platelet Rich Fibrin-A comparative study in Mandibular Third Molar Impaction sockets. J Oral Maxillofac Surg 75(7):1322–1329. 10.1016/j.joms.2017.01.035 28249808 10.1016/j.joms.2017.01.035

[31] Baslarli O, Tumer C, Ugur O, Vatankulu B (2015) Evaluation of osteoblastic activity in extraction sockets treated with platelet-rich fibrin. Med Oral Patol Oral Cir Bucal 20(1):e111–e116. 10.4317/medoral.19999 25475771 10.4317/medoral.19999 PMC4320413

[32] Kaya S, Koç A (2022) Is there an association between proton pump inhibitors and radiomorphometric parameters of the mandible? A preliminary study. Oral Radiol 38(4):586–593. 10.1007/s11282-022-00593-3 35119594 10.1007/s11282-022-00593-3

[33] Schneider CA, Rasband WS, Eliceiri KW (2012) NIH Image to ImageJ: 25 years of image analysis. Nat Methods 9(7):671–675. 10.1038/nmeth.2089.c 22930834 10.1038/nmeth.2089 PMC5554542

[34] Schindelin J, Rueden CT, Hiner MC, Eliceiri KW (2015 Jul-Aug) The ImageJ ecosystem: an open platform for biomedical image analysis. Mol Reprod Dev 82:7–8. 10.1002/mrd.22489 10.1002/mrd.22489 PMC5428984 26153368

[35] Alzahrani AA, Murriky A, Shafik S (2017) Influence of platelet rich fibrin on post-extraction socket healing: a clinical and radiographic study. Saudi Dent J 29(4):149–155. 10.1016/j.sdentj.2017.07.003 29033524 10.1016/j.sdentj.2017.07.003 PMC5634795

[36] Temmerman A, Vandessel J, Castro A, Jacobs R, Teughels W, Pinto N, Quirynen M (2016) The use of leucocyte and platelet-rich fibrin in socket management and ridge preservation: a splitmouth, randomized, controlled clinical trial. J Clin Periodontol 43(11):990–999. 10.1111/jcpe.12612 27509214 10.1111/jcpe.12612

[37] Pereira DA, Mendes PGJ, de Souza Santos S, de Rezende Barbosa GL, Pessoa RSE, de Oliveira GJPL (2022) Effect of the association of infra-red and red wavelength photobiomodulation therapy on the healing of post-extraction sockets of third lower molars: a split-mouth randomized clinical trial. Lasers Med Sci 37(5):2479–2487. 10.1007/s10103-022-03511-5 35079918 10.1007/s10103-022-03511-5

[38] Joensen J, Demmink JH, Johnson MI, Iversen VV, Lopes-Martins RÁ, Bjordal JM (2011) The thermal effects of therapeutic lasers with 810 and 904 nm wavelengths on human skin. Photomed Laser Surg 29(3):145–153. 10.1089/pho.2010.2793 21219241 10.1089/pho.2010.2793

[39] Stamborowski SF, de Oliveira Spinelli BM, Lima FPS, Costa DR, de Silveira Souza GA, Lima MO, Lopes Martins RAB (2021) The influence of photobiomodulation on the temperature of the brachial biceps during muscle fatigue protocol. Lasers Med Sci 36(8):1741–1749. 10.1007/s10103-021-03360-8 34255219 10.1007/s10103-021-03360-8

